# 5-Methylcytosine Methylation-Linked Hippo Pathway Molecular Interactions Regulate Lipid Metabolism

**DOI:** 10.3390/ijms26062560

**Published:** 2025-03-12

**Authors:** Lichen Du, Rui Gao, Zhi Chen

**Affiliations:** 1 Agricultural College, Yangzhou University, Yangzhou 225009, China; cicydlc@outlook.com; 2 College of Animal Science and Technology, Yangzhou University, Yangzhou 225009, China

**Keywords:** 5mC, hippo pathway, lipid metabolism, co-regulation

## Abstract

5-methylcytosine (5mC) is a common form of DNA methylation, essentially acting as an epigenetic modification that regulates gene expression by affecting the binding of transcription factors to DNA or by recruiting proteins that make it difficult to recognize and transcribe genes. 5mC methylation is present in eukaryotes in a variety of places, such as in CpG islands, within gene bodies, and in regions of repetitive sequences, whereas in prokaryotic organisms, it is mainly present in genomic DNA. The Hippo pathway is a highly conserved signal transduction pathway, which is extremely important in cell proliferation and death, controlling the size of tissues and organs and regulating cell differentiation, in addition to its important regulatory roles in lipid synthesis, transport, and catabolism. Lipid metabolism is an important part of various metabolic pathways in the human body, and problems in lipid metabolism are related to abnormalities in key enzymes, related proteins, epigenetic inheritance, and certain specific amino acids, which are the key factors affecting its proper regulation. In this article, we will introduce the molecular mechanisms of 5mC methylation and the Hippo signaling pathway, and the possibility of their co-regulation of lipid metabolism, with the aim of providing new ideas for further research and novel therapeutic modalities for lipid metabolism and a reference for the development and exploration of related research.

## 1. Introduction

**Figure 1:**
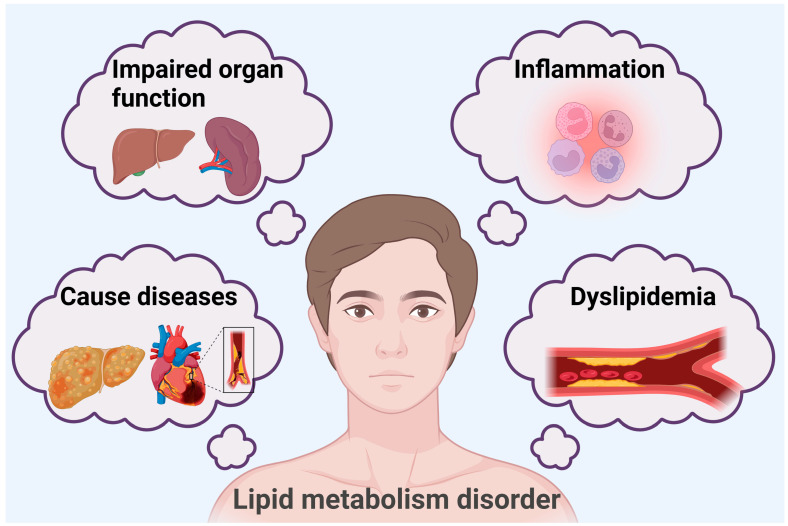
Lipid metabolism disorder-induced changes in the body.

Lipids, which are essential biomolecules, are important forms of energy storage in living organisms and are indispensable for maintaining the stability of cell membranes. In addition, lipid molecules are involved as signaling molecules in the human body for a variety of pathological functions [1]. Lipid metabolism is a complex and multilayered regulatory process. Considering the regulation of body lipid storage through selective autophagy as an example, lipophagy mediated by Rab GTPase, enzymes, ion channels, and transcription factors can reduce liver lipid content [2], regulate pancreatic lipid metabolism abnormalities [3], and regulate adipose tissue differentiation [4], thus maintaining the stability of body lipid content. Abnormal lipid metabolism disturbs fat processing in the body, which can lead to an abnormal accumulation of or reduction in lipids and their metabolites in the blood and tissues. A series of bodily changes may be triggered when there is an abnormality of lipid metabolism in the body. First, there will be abnormal blood lipid levels [5] and enhanced inflammatory responses [6,7], and long-term lipid metabolism disorders result in the impaired function of related organs [8]. Abnormalities in lipid metabolism can also lead to related diseases, including hyperlipidemia [9], nonalcoholic fatty liver [10], metabolic dysfunction-associated steatotic liver disease [9,11], and cardiovascular disease [12]. Relevant studies have also shown that fat transfer between brain cells in Alzheimer’s disease leads to the accumulation of lipid droplets in microglia, which triggers inflammatory responses and phagocytic dysfunction in microglia [13] (Figure 1).

5-methylcytosine(5mC) methylation is a common DNA modification in eukaryotes, which can modify gene promoter regions and coding regions, thus repressing gene regulation and expression. 5mC methylation in lipid metabolism and the methylation of related factors and enzymes affect its metabolic stability [14]. The Hippo pathway is a signaling pathway that mainly acts at the cellular and tissue levels, and through the regulation of lipid cells and lipid synthesis, it is able to influence the lipid storage and metabolism of living organisms. 5mC methylation and related factors in the Hippo pathway interact with each other [15,16]. Complexes formed by effectors in the Hippo pathway affect the expression of transferases during 5mC methylation. As 5mC methylation and the Hippo pathway affect lipid metabolism, and the two pathways are also closely related, this essay will focus on the regulation of lipid metabolism through the interaction of 5mC methylation and the Hippo pathway and then propose more possibilities for solving the problem of lipid metabolism.

## 2. Mechanisms and Influencing Factors of 5mC Methylation

### 2.1. Introduction to 5mC Methylation

**Figure 2:**
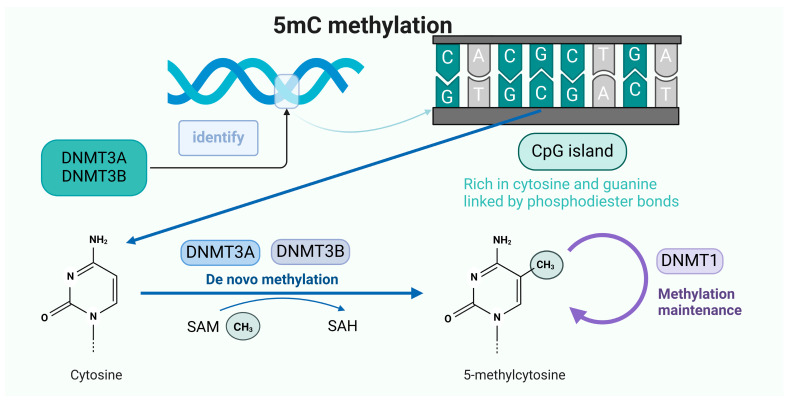
The occurrence mechanism of 5mC methylation.

5mC methylation occurs when a chemical modification occurs in the cytosine base in the DNA molecule. Normal cytosine is composed of a pyrimidine ring that contains carbon, hydrogen, and nitrogen atoms. When 5mC methylation occurs, a methyl (-CH_3_) group is added to the fifth carbon atom of cytosine, which changes its chemical structure, resulting in changes in properties such as spatial structure and electron distribution (Figure 2).

In 1925, Johnson and Coghill first isolated nucleic acids from Mycobacterium tuberculosis and discovered 5mC [17]. In 1965, Arber proposed a restriction modification system in which methylation-sensitive restriction enzymes protect the bacteria from invasion by bacteriophage DNA, and the DNA of the bacteria is protected from enzyme cutting due to methylation [18]. In 1969, Srinivasan and Borek speculated about and tested the methylation capacity of different species, suggesting that different tissues of the same organism might have different 5mC contents [19]. In 1985, Bird and colleagues discovered that hypomethylated extended regions in spermatid consisted of CpG-rich DNA [20], which were later popularized under the name CGIs (CpG islands). In 1993, Finnegan and Dennis identified and cloned the first plant DNA methyltransferase (DNMT), MET1, using sequence homology with mouse and bacterial methyltransferases [21].

The de novo methylation methyltransferases DNMT3A and DNMT3B recognize CpG dinucleotide sequences in DNA sequences and, after identifying the methyl site, transfer the methyl group on SAM to cytosine to form 5mC, which is used to maintain methylation during cell proliferation using DNMT1.

### 2.2. Mechanism of 5mC Methylation

DNA methyltransferases (DNMTs) are key to 5mC methylation and are responsible for catalyzing the conversion of cytosine to 5mC by S-Adenosyl-L-Methionine Disulfate Tosylate (SAM) as a methyl donor; currently, the main known DNMTs include DNMT1, DNMT2, DNMT3A, DNMT3B, and DNMT3L. Unlike DNMT1, DNMT3A, and DNMT3B, DNMT3L does not have any enzymatic activity, but increases the catalytic activity of DNMT3A and DNMT3B [22]. DNMT1 is responsible for maintaining the existing methylation pattern, and DNMT3A and DNMT3B are responsible for participating in de novo methylation [23]. DNMT1 is a large multi-structural-domain enzyme consisting of approximately 1620 amino acids, which maintains the established methylation pattern during replication by copying existing methyl tags to the nascent DNA strand during cell division [24]. Researchers have found that a loss of DNMT1 from the parent nucleus during somatic cell nuclear transfer results in incorrect imprinted methylation, a finding that supports the critical role of DNMT1 in the maintenance of methylation [25]. DNMT3A and DNMT3B, as de novo methylation methyltransferases, are essential for the completion of methylation, especially in the establishment of new methylation patterns. Their main function is the de novo methylation modification of unmethylated DNA double strands [26,27], and the significance of the de novo methylation of DNMT3A and DNMT3B is particularly reflected in early embryonic development, where DNMT3A and DNMT3B in vivo have common functions and target specificity and are involved in the early embryonic development of the Rasgrf1 DMR and the long terminal repeat IAP and Line1 methylation [28].

Methyl donors are compounds that can provide methyl (-CH3) groups, and SAM is one of the most important physiologically active methyl donors, synthesized in a process where methionine adenosyltransferase catalyzes methionine, which is produced by the one-carbon metabolic cycle [29]. SAM not only serves as a direct methyl donor, but also indirectly promotes other methyltransferase functions, in addition to which SAM metabolites may influence the link between the mTOR signaling pathway and DNA methylation [29,30].

In addition to DNMTs and SAM, 5mC methylation is another important component of CGIs, which are regions of the genome enriched in cytosine–phosphate–guanine dinucleotides (CpG dinucleotides). CGIs are usually located in the promoter or first exon region of genes and are typically a few hundred to a few thousand base pairs in length, which generally remain in the unmethylated state [31]; if aberrant methylation occurs, it may lead to changes in gene expression [20,32,33]. Some studies have indicated that the aberrant methylation of CGIs leads to the silencing of gene expression [34]; for example, the hypermethylation of CGIs of both Runx3 and MT-3 has been found to be associated with cancer [35,36]. In carcinoma of the colon and rectum, the hypermethylation of the promoter CGI of the mismatch repair gene MLH1 causes the transcriptional inactivation of the gene [37]. It has also been suggested that the inactivation of the tumor suppressor gene PTEN may result from the aberrant methylation of the CGI in the promoter region [38,39]. In summary, the study of CGIs is extremely important in the study of methylation regulation. Methylated CpG sites in these regions attract enzymes that can continue to methylate other CpG sites, where unmethylated CpG sites attract demethylating enzymes that can retain their collective state over multiple cellular generations while continuously maintaining the alternative methylation state [40]. The methylation of CGIs usually suppresses gene expression when there are no DNMTs present to add methyl groups to the cytosine of the CGIs. CGIs assume a state of normal gene expression, that is, the demethylation of CGIs, which is dependent on high densities of nonmethylated CpG dinucleotides [41]. In both humans and mice, approximately 60% of promoters co-localize with CGIs, and these regions are unmethylated and have higher G and C contents than the entire genome [42]. The methylation of CGIs recruits a variety of proteins, and these recruited proteins play important roles in the regulation of gene expression; among the recruited proteins are histone deacetylases (HDACs) [42], histone methyltransferases (HMTs) [43], DNA methyltransferases [44,45], and polycomb group protein complexes [46], with their specific functions listed in the table below (Table 1).

5mC plays a role in the regulation of gene expression, cell differentiation, genome stability, and disease development. 5mC can repress the regulation of the expression of relevant genes by affecting the chromatin structure and binding of transcription factors, and a high density of 5mC methylation in the promoter region silences genes [47] and also promotes the progression of cellular development by altering the activity of specific genes through the modification of 5mC methylation [48,49]. During cell differentiation and development, changes in 5mC methylation levels can maintain pluripotency or promote the generation of specific types of cells as embryonic stem cells differentiate into different lineages [43,50]. In existing studies, 5mC methylation has also been found to be closely associated with genome stability as an important DNA modification that maintains genome stability in organisms in a variety of ways, including regulating the structure and expression of chromatin, participating in the DNA repair process, and responding to oxidative stress [51,52,53,54]. 5mC methylation has also been associated with diseases and has been shown to be a key factor in the development of aging-associated brain diseases, such as Alzheimer’s disease. 5mC methylation has been found to be potentially involved in neuronal activity and supporting structures, which aberrant DNA methylation patterns may interfere with, therefore promoting neuronal death [55]. In addition, the abnormal distribution of 5mC may lead to the malignant transformation of cells, which is a major factor in the formation of tumors [47,56]. With the development of more technologies, current research on 5mC methylation is moving toward multi-dimensional and multi-level development, and it is hoped that the further optimization of detection technology will reveal more specific functions and the potential application value of 5mC methylation in various fields.

### 2.3. Factors Influencing 5mC Methylation

The molecular mechanisms and functional roles of 5mC methylation are discussed above, so what are the factors that affect the occurrence of 5mC methylation? The factors affecting 5mC methylation are very complex, and in recent years, some researchers have found significant changes in methylation in the region of the gene body through whole-genome methylation sequencing by studying the care behavior of beetles from different generations [57]. It is not difficult to see the possibility that genetic factors may affect the level of 5mC methylation. In plant research using tea trees as experimental materials, some studies have found that seasonal changes in the environment cause genome-wide changes in 5mC methylation in the new shoots of tea trees [58], and others have demonstrated that in different tissues of the tea tree, the levels of 5mC methylation are significantly different [59]. MenSCs affect the distribution of 5mC and 5hmC in the vicinity of hepatocellular carcinoma, increase the level of 5-hmC in the vicinity of oncogenes, and, at the same time, decrease the level of 5-hmC in the vicinity of pro-carcinogenic genes [60]. Changes in the levels of 5mC and 5hmC are also present in the brain cells, and in the normal development of the brain, DNA methylation patterns change over time and with environmental influences, whereas in a diseased state, aberrant DNA methylation may lead to the dysregulated expression of certain genes, thus triggering neuronal cell dysfunction [50]. These studies indicate that 5mC methylation is affected by both environmental and physiological factors. In the elaboration of the role of 5mC methylation outlined above, it was mentioned that 5mC methylation leads to disease development; in contrast, disease factors also affect 5mC content. For example, in chronic kidney disease, the level of 5mC methylation in renal tubular epithelial cells is positively correlated with the degree of renal fibrosis; researchers have found that the hypermethylation of the promoter of the HOXA5 gene leads to a reduction in its expression, which promotes renal fibrosis development [61]. In addition, some biotechnological tools also affect the occurrence of 5mC methylation, as when analyzing DNA methylation, different experimental technological means and tools can bring about different disturbances; for example, when the ELISA method is used to assess 5mC and 5hmC levels globally, it may be biased due to differences in sample handling or reagents [62]. In the study of 5mC methylation, there are many influencing factors, and it is still necessary to explore the causes and results of 5mC methylation changes in various organisms with the goal of reducing the differences in content levels caused by experimental techniques and tools to gain a deeper understanding of the core mechanism of 5mC methylation.

## 3. Introduction of and Molecular Mechanisms Related to the Hippo Pathway

The Hippo signaling pathway, which mainly regulates organ size and tissue homeostasis, was initially discovered in Drosophila melanogaster by genetic mosaic screening as a key factor for tissue growth, and has been imaginatively referred to as the Hippo pathway, as its mutants exhibit abnormally large organ morphology with an appearance similar to that of a hippopotamus [63].

**Figure 3:**
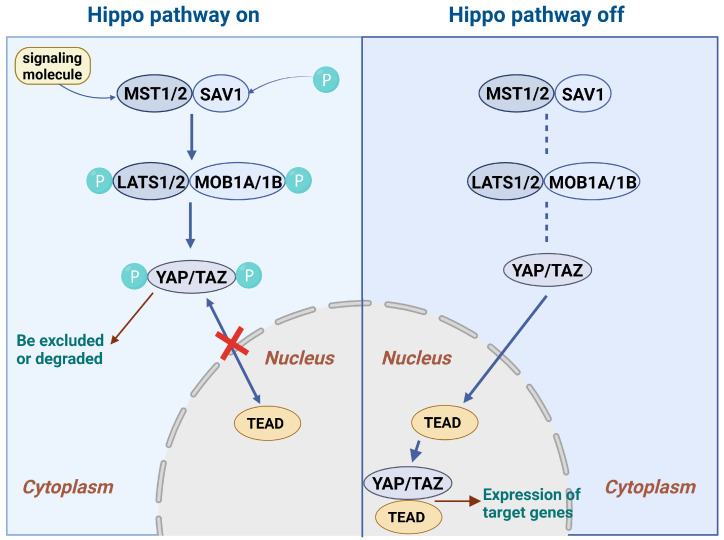
The progression of the HIPPO signaling pathway.

The molecular composition of the Hippo pathway includes a core kinase cascade response molecule, adaptor protein, and transcriptional coactivator. Among these, the core kinase cascade complex consists of several key molecules, including MST1/2 (the mammalian homolog of Hippo in mammals) and LATS1/2 (the mammalian homolog of Warts in Drosophila). The activation of the Hippo pathway can be triggered by a variety of extracellular signals and changes in the intracellular state. The inhibition of intercellular contact is an important activator, and the Hippo pathway is activated when cell density increases and cells come into greater contact with each other. In addition, changes in the extracellular matrix, mechanical stress, and growth factor deficiency can also initiate the pathway [64,65]. MST1/2 can be activated by upstream signals, MST1/2 is able to phosphorylate and activate downstream molecules, and LATS1/2, which is a direct downstream molecule that receives phosphorylation signals from MST1/2, is activated [66]. Adaptor proteins also include SAV1 and MOB1. SAV1 is the mammalian homolog of Salvador in Drosophila, which can interact with MST1/2 and promote the activation and stabilization of MST1/2. MOB1 is an important auxiliary protein in LATS1/2. MOB1 binds to LATS1/2, enhances the kinase activity of LATS1/2, and is involved in the substrate recognition and phosphorylation of LATS1/2 [66,67,68,69]. Transcription coactivator molecules include YAP and TAZ, which are the main effector molecules; YAP/TAZ can enter the nucleus and interact with transcription factors such as TEAD (TEA-domain family members) to initiate the transcription of a series of genes. When the Hippo pathway is not activated, YAP/TAZ is dephosphorylated and can stably exist in the nucleus, promoting the expression of cell proliferation-related genes. When the Hippo pathway is activated, YAP/TAZ is phosphorylated, resulting in the exclusion of YAP/TAZ from the nucleus or degradation of YAP/TAZ, which inhibits the expression of cell proliferation-related genes [70,71,72]. In addition, the Hippo pathway involves a number of other auxiliary proteins, such as the GC kinase Hippo, which makes up the core pathway components and phosphorylates the non-catalytic peptide Mats/Mob1 in the presence of the scaffold protein Salvador [73,74]. (Figure 3).

The Hippo signaling pathway has many important biological functions; it can determine organ size by balancing cell proliferation and apoptosis, and it can control the size of Drosophila or the regeneration of the mammalian liver [75,76]. In addition, the Hippo pathway is also involved in the regulation of organ size, stem cell self-renewal, and tumor suppression [77,78].

After activation by signaling molecules, MST1/2 forms a complex with SAV1 and phosphorylates it, activating the downstream LATS1/2, which binds to MOB1 and enhances its activity. When the Hippo signaling pathway is activated, LATS1/2 and MOB1 phosphorylate YAP/TAZ, which is excluded from the nucleus or degraded. When the Hippo signaling pathway is not activated, YAP/TAZ is in a dephosphorylated state and can stably exist in the nucleus.

## 4. The Role of 5mc Methylation and the Hippo Pathway in the Regulation of Lipid Metabolism

### 4.1. Regulation of Lipid Metabolism by 5mc Methylation

In the previous sections, we mentioned various functions of 5mC in gene expression regulation, cell differentiation, genome stability, and disease occurrence, and we specifically described the regulation of 5mC in methylation in mammalian hepatic lipid metabolism. Relevant studies have shown that in the regulation of gene expression, 5mC affects the hydroxymethylation of β-oxidation genes in the liver, which in turn induces hepatic lipid accumulation and impaired glucose metabolism. 5mC affects intracellular lipid levels by altering the methylation status of specific genes, which, depending on the level of methylation, can repress or activate genes closely related to lipid metabolism [79,80]. In terms of lipid synthesis, 5mC can repress the expression of genes related to lipid metabolism and indirectly promote an increased lipid content by interacting with other epigenetic modifications. For example, the reduced methylation of the promoter region of the sterol regulatory element-binding protein 1 (SREBP-1c) gene and its upregulation in stem cells activate the expression of genes related to fatty acid synthesis and lipid transport, which indirectly results in the expression of genes related to hepatic lipid synthesis and lipid transport [79,80], which indirectly leads to lipid accumulation in hepatocytes [81].

At present, the study of 5mC methylation has made some progress in liver lipid metabolism, adipocytes, and methyl nutrient-related aspects; however, the specific mechanism is not yet clear, and further research is needed to determine this. Meanwhile, the authors believe that the mechanism of 5mC methylation in lipid metabolism can be studied in depth to further clarify the specific mechanism of 5mC methylation in different cell types for the regulation of lipid metabolism, for example, in liver nonparenchymal cells and different types of adipocytes, and whether there are other pathways, in addition to the known pathways, and targets of 5mC methylation in the regulation of lipid metabolism. Through the use of a technical system of multi-omics association, the dynamic network of relationships between 5mC methylation and lipid metabolism-related genes, proteins, and metabolites will be more comprehensively analyzed, and new regulatory factors and modes of regulation will be identified.

### 4.2. Regulation of Lipid Metabolism by Hippo Pathway

The Hippo signaling pathway regulates lipid metabolism from beginning to end, with the expression of lipid synthesis-related genes, inhibition of adipocyte differentiation and proliferation, regulation of adipokine secretion, and influence of energy metabolism in the liver and adipose tissue, and finely regulates lipid metabolism at multiple levels and from multiple angles, thus maintaining the balance and homeostasis of lipid metabolism in the body.

In maintaining the balance of lipid metabolism, YAP/TAZ acts through two mechanisms. TAZ can directly inhibit the function of PPARγ, which in turn reduces the expression of genes related to adipogenesis and lipid storage, whereas YAP/TAZ, by binding to the TEAD transcription factor, directly acts on the enhancer region upstream of the leptin gene to upregulate its expression, and the increase in the level of leptin can help to increase energy expenditure, prevent lipotoxicity, and maintain metabolic homeostasis. During signal transduction and gene expression, when the Hippo pathway is inactivated, the YAP nucleus is translocated and binds to the TEAD nuclear transcription factor, thus regulating the expression of downstream genes and ultimately participating in lipid metabolism [82,83]. In addition, SREBP-dependent lipid synthesis promotes tumor cell proliferation, and SREBP can act by enhancing the activation of effector molecules downstream of the Hippo signaling pathway [84]. In addition to the direct regulation of lipid synthesis, the Hippo pathway interacts with other metabolic pathways, such as the mTOR signaling pathway, which in some cases inhibits amino acid and lipid metabolism; this regulation is particularly important in tumor cells [85,86]. Cell membrane lipid peroxidation is one of the key mechanisms to induce iron death, which activates the cGAS-STING pathway, wherein GPX4 inactivation leads to the accumulation of lipid peroxides, which ultimately triggers iron death; the Hippo pathway may also be involved as a signaling node responding to the generation of excessive ROS, thus exerting its influence during lipid metabolism [87,88,89]. The mechanism of the Hippo pathway in lipid metabolism is relatively more mature; however, lipid metabolism requires multi-tissue joint participation, and the Hippo signaling pathway is an important linking bridge in the organism. The further study of how this bridge mediates the signaling exchange between different organs and tissues to jointly regulate lipid metabolism can help determine, for example, whether the Hippo pathway is used as a signaling node between the liver and adipose tissue, and between the muscle and adipose tissue. Whether lipid synthesis, storage, and catabolism are coordinated between the liver and adipose tissue, and muscle and adipose tissue, through Hippo pathway-dependent signaling molecules can be investigated, thus exploring how the Hippo signaling pathway is involved in the whole-body lipid metabolism regulatory network.

### 4.3. Interaction Between 5mC Methylation and Hippo Pathway Affects Lipid Metabolism

5mC methylation and the Hippo pathway each play a role in lipid metabolism, and there is a lack of research on the interaction between the two. Based on the few studies that have been conducted, it can be hypothesized that 5mC methylation can inhibit the expression of key genes and related lipid metabolism genes in the Hippo pathway by altering the methylation status of gene expression regulatory elements, while the Hippo pathway can alter the methylation status of gene expression regulatory elements through signal transduction, thus affecting the expression of key genes and related lipid metabolism genes. The Hippo pathway may alter the activity or localization of methylation-related enzymes through signaling, and these two intertwine to regulate lipid synthesis, storage, and catabolism at multiple levels, ultimately affecting lipid metabolism homeostasis.

**Figure 4:**
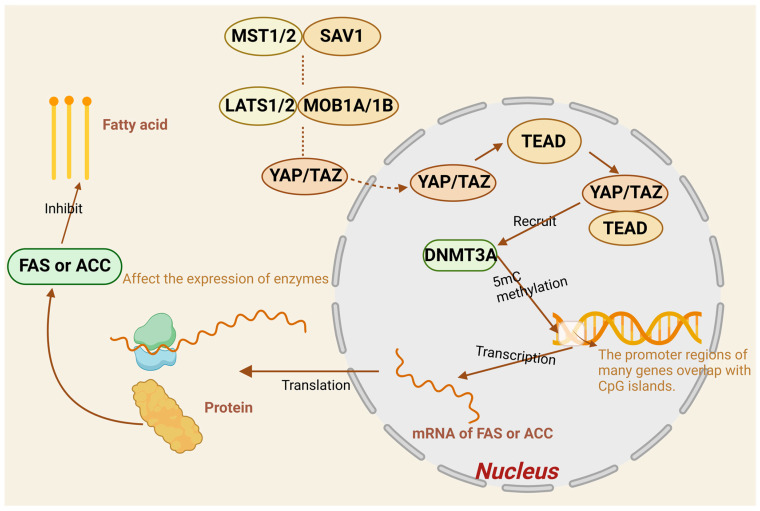
Factors in the Hippo pathway influence 5mC methylation to regulate fatty acid formation.

It has been pointed out that there is direct interaction between DNMT3A and YAP/TAZ, and researchers have controlled the expression of DNMT3A and YAP/TAZ and their translocation effects on the gallbladder through cellular and animalistic experiments and found that DNMT3A expression was significantly upregulated, suggesting that DNMT3A can be recruited by YAP/TAZ to the promoter regions of specific genes, thereby promoting methylation [90]. According to the above, DNMT3A is a key enzyme of methylation and can catalyze methylation; for example, the **anti-oncogene** CDH1 promoter can inhibit gene expression and promote the proliferation, invasion, and metastatic ability of tumor cells by recognizing CGIs and performing DNA methylation modification [90,91]. Altogether, we conclude that DNMT3A, the key enzyme of methylation, is likely to be recruited by YAP/TAZ in the Hippo signaling pathway, which catalyzes the onset of 5mC methylation under certain conditions and ultimately raises the level of methylation, thus suppressing the expression of relevant genes to affect the relevant tissues. In the whole process of fat metabolism, it is assumed that the promoter region of DNMT3A of enzymes and proteins with a variety of roles is recruited more due to and presents high methylation levels, which can inhibit the action of enzymes and proteins, thus repressing normal lipid metabolism. For example, fatty acid synthase is a central regulator of lipid metabolism and promotes the expression of other key genes in the lipogenesis pathway [92,93]. Acetyl-CoA carboxylase can enhance the rate of fatty acid synthesis and fatty acid content in organisms and reduce lipid accumulation by regulating the AMPK/PPARα/CPT1A pathway [94,95] and is a key factor in lipid metabolism. Therefore, we hypothesized that the promoter region of genes such as fatty acid synthase and acetyl coenzyme A carboxylase could increase the content of DNMT3A in cells through the interaction of DMTA3A and YAP/TAZ and methylation modification in the promoter region, which could inhibit the expression of the two enzymes, inhibit lipogenesis, and achieve the purpose of regulating lipid metabolism (Figure 4).

Unphosphorylated YAP/TAZ enters the nucleus and forms a complex with TEAD that recruits DNMT3A. Most genes have an overlapping region between the promoter and CGI, and the recognition of the overlapping sequences on the promoter by DNMT3A catalyzes the methylation of 5mC, which affects the activity of the promoter, and ultimately represses the expression of fatty acid synthetase (FAA) and acetyl coenzyme A carboxylase (ACC), thereby inhibiting fatty acid formation.

Researchers have identified TET1, a 5-methylcytosine dioxygenase, as a direct target of YAP, and if YAP activation induces the expression of TET1, TET1 physically interacts with TEAD accordingly, forming a complex that can influence subsequent epigenetic regulation and transcriptional activation, and YAP, an effector of the Hippo signaling pathway, promotes DNA methylation reprogramming through TeT1-mediated DNA methylation to promote liver cell proliferation and hepatocarcinogenesis [15], which provides support for the hypothesis that YAP and methylation interact. The demethylation process has been divided into active and passive methylation [96], and the TET protein family is an important protein in active demethylation, which first oxidizes 5mC to 5hmC [97] and then further oxidizes 5hmC to 5fC and 5caC, with the two products generated being removed by Thymine-DNA Glycosylase. These two products are directly removed by passive dilution or TDG and other base excision repair enzymes, which ultimately completes the demethylation process [98,99]. The TeT1 protein is able to mediate the process of active demethylation, and if activated by YAP, it can induce the increased expression of TET1, which enhances the degree of DNA demethylation. Accordingly, the authors would like to put forward a hypothesis; from the previous paragraph, it is known that in the process of fatty acid synthesis, fatty acid synthase and acetyl-CoA carboxylase are enzymes with key roles, the activation of YAP in the Hippo signaling pathway can induce the overexpression of TET1, and the binding of TET1 to the promoter region of the corresponding enzyme can promote demethylation, reduce the methylation level in the cell, and promote fatty acid synthase and acetyl-CoA carboxylase expression, thus increasing fatty acid synthesis. PPARγ is a “major regulator” of adipogenesis, which influences adipocyte differentiation and maturation and maintains adipocyte life [100,101]. If TET1 binds to its promoter and promotes its expression, it can further affect lipid storage and metabolism in adipocytes. The insulin signaling pathway is an important pathway for lipid catabolism [102,103], and TET1 can indirectly stimulate lipid uptake, synthesis, and catabolism by combining with the epigenetic regulation of key molecular genes in the insulin signaling pathway. Tet1 may also affect the methylation status of insulin receptor substrate (IRS) genes, altering the expression and function of IRS proteins, thus promoting the activity of downstream lipid metabolism-related kinase activities and ultimately regulating the balance of intracellular lipid metabolism.

In addition, some researchers have found that leptin, a hormone secreted by adipose tissue, can inhibit the expression of TET2 through the JAK2/STAT3 signaling pathway, while TET2 reduces the methylation level of the leptin promoter and promotes the expression of the leptin gene by interacting with the transcription factor C/EBPα. TET2 can control body weight through the modulation of food intake and energy expenditure [104]. JAK2/STAT3 is also an intracellular signaling pathway that plays an important role in cell growth, differentiation, apoptosis, and immune regulation. In recent years, researchers of the shrimp Hippo signaling pathway and immune response have mentioned that the Hippo signaling pathway and STAT and other immune-related transcription factors have a complex regulatory relationship [105], which also implies that the JAK2/STAT3 and Hippo signaling pathways interact with each other in some aspects; therefore, whether there is also a role of TET2 in regulating the balance of lipid metabolism between these two pathways requires further investigation.

## 5. Discussion and Outlook

Several studies have shown that 5mC methylation and the Hippo pathway play important roles in lipid metabolism. 5mC methylation precisely regulates the expression of genes related to lipid synthesis, transport, and catabolism at the transcriptional level through the methylation modification of those genes, which in turn affects the production, transport, and catabolism rates of lipids and maintains intracellular lipid homeostasis. The Hippo pathway senses changes in intracellular and extracellular environments and regulates the activity and localization of the key effectors YAP/TAZ by means of the kinase cascade reaction among its core members, thus closely controlling adipocyte differentiation, lipid production, and storage. Although the dynamic regulation of the Hippo signaling pathway and 5mC methylation is still unclear and there is a lack of relevant studies, the interaction between the two can be observed in existing experiments, and it is possible that the two form a complex regulatory network through their interactions in the process of lipid metabolism. In the future, we hope to further understand the dynamic changes in the whole regulatory network, study the interaction between 5mC methylation and the Hippo pathway in different cells through single-cell sequencing and whole-genome methylation sequencing, and study the intrinsic connection and regulation between the two in depth.

**Figure 5:**
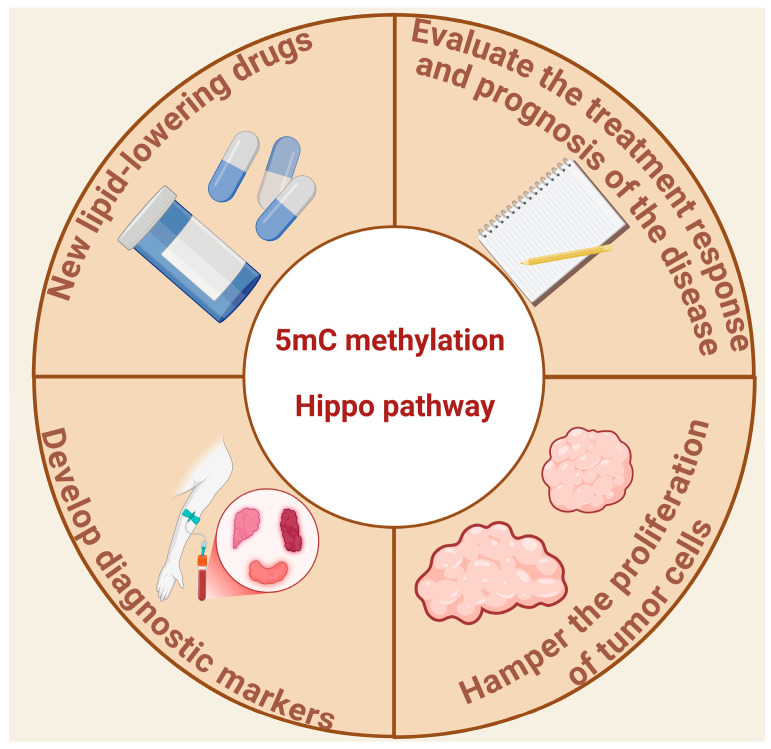
Practical future applications of studying the interaction of 5mC methylation and the Hippo pathway.

The study of the relationship between the two factors and the prevention and treatment of many lipid diseases, such as obesity and fatty liver, may provide a theoretical basis for the development of new therapeutic targets and strategies, and provide ideas and potential breakthroughs for the development of novel lipid-lowering drugs. To carry out personalized treatment and precise diagnosis for lipid metabolic diseases with family genetic predisposition, early prevention and early screening may be achievable through methylation profiles and Hippo pathway-related gene mutation and content status. Clinically, it may be possible to develop more sensitive and specific diagnostic markers for lipid metabolism disorders and related diseases based on the common detection indexes of 5mC methylation and the Hippo pathway, such as through the level of 5mC methylation of specific genes in blood or tissues, and the expression or activity of key proteins of the Hippo pathway, which can be used to diagnose lipid metabolism abnormalities and predict the risk of disease at an early stage, and to realize early-stage disease intervention and treatment. Some researchers have suggested that reducing fatty acids can inhibit the proliferation of tumor cells [106], and through the study of 5mC methylation and the Hippo pathway, it may be possible to prevent and diagnose other diseases by regulating fatty acids to inhibit the proliferation of specific tumor cells in the future, thus opening up a new avenue of development in therapeutic research on tumors and other diseases. In addition to prevention and treatment, the measurement of 5mC methylation and Hippo pathway content may also provide new possibilities for disease assessment, and the detection of both in patients, compared with normal levels, can help assess the response to treatment and the prognosis of the disease. Therefore, the synergistic regulation of 5mC methylation and the Hippo pathway has potential and developmental prospects and is worthy of further exploration (Figure 5).

## Figures and Tables

**Table 1:** Functions of scaffold proteins.

Protein Names	Functions
Histone deacetylase (HDAC)	1. Catalyzes histone deacetylation, preventing transcription factors from binding to DNA and inhibiting gene expression. 2. Recruits other repressors to enhance gene expression inhibition. 3. Regulates specific gene expression during cell differentiation and development.
Histone methyltransferase (HMT)	1. Modifies methylation of specific amino acid residues of histones. 2. Alters chromatin structure and function, affecting binding of transcription factors. 3. Dynamically regulates gene expression levels and participates in a variety of cellular biological processes.
DNA methyltransferase (DNMT)	1. DNMT1 maintains methylation state during DNA replication and ensures stable inheritance of methylation patterns. 2. DNMT3A and DNMT3B carry out de novo methylation to establish new methylation patterns in early embryonic development and promote cell differentiation. 3. DNA methylation directly or indirectly represses gene transcription.
Polycomb group protein complex—PRC1	1. Inhibit gene expression. 2. Promotes the compression of chromatin fibers, making it difficult for gene promoters to be approached by transcription factors and achieving gene silencing.
Polycomb group protein complex—PRC2	1. Generates gene silencing signatures. 2. Enhances gene expression inhibition.
